# Hospital-acquired influenza in an Australian tertiary Centre 2017: a surveillance based study

**DOI:** 10.1186/s12890-019-0842-6

**Published:** 2019-04-16

**Authors:** Nikita Parkash, Wendy Beckingham, Patiyan Andersson, Paul Kelly, Sanjaya Senanayake, Nicholas Coatsworth

**Affiliations:** 1 Department of Infectious Diseases, Canberra Hospital and Health Services, Canberra, Australian Capital Territory Australia; 2 Infection Prevention and Control, Canberra Hospital and Health Services, Canberra, Australian Capital Territory Australia; 3 National Centre for Epidemiology and Population Health, Australian National University, Canberra, Australian Capital Territory Australia; 4 Australian National University Medical School, Canberra, Australian Capital Territory Australia; 5 Population Health and Prevention Division, ACT Health, Canberra, Australian Capital Territory Australia

**Keywords:** Cross infection, Disease transmission, Infectious, Infection control, Influenza, Human, Sentinel surveillance

## Abstract

**Background:**

In 2017, Australia experienced its highest levels of influenza virus activity since the 2009 pandemic. This allowed detailed comparison of the characteristics of patients with community and hospital-acquired influenza, and infection control factors that contributed to influenza spread.

**Methods:**

A surveillance based study was conducted on hospitalised patients with laboratory-confirmed influenza at the Canberra Hospital during April–October 2017. Differences between the hospital-acquired and community-acquired patient characteristics and outcomes were assessed by univariate analysis. Epidemiologic curves were developed and cluster distribution within the hospital was determined.

**Results:**

Two hundred and ninety-two patients were included in the study. Twenty-eight (9.6%) acquired influenza in hospital, representing a higher proportion than any of the previous 5 years (range 0.9–5.8%). These patients were more likely to have influenza A (*p* = 0.021), had higher rates of diabetes (*p* = 0.015), malignancy (*p* = 0.046) and chronic liver disease (*p* = 0.043). Patients acquiring influenza in hospital met clinical criteria for influenza like illness in 25% of cases, compared with 64.4% for community-acquired cases (*p* < 0.001). Hospital-acquired influenza cases occurred in two distinct clusters. Patients were moved an average of 5 times after diagnosis. Mean length of stay following diagnosis was 13 days compared to 5 days for community-acquired cases (*p* < 0.001). Of the patients with hospital-acquired influenza, 22 were in shared rooms during their incubation period and 9 were not isolated in single rooms following diagnosis. Treatment was initiated within the recommended 48 h period following symptom onset for 62.5% of hospital-acquired cases compared with 39.8% of community-acquired cases (*p* = 0.033).

**Conclusions:**

Our results show that clinical presentation differed between patients with hospital-acquired influenza compared with those who acquired influenza in the community. Cases occurred in two clusters suggesting intra-hospital transmission rather than random importation from the community, highlighting the importance of infection control measures to limit influenza spread. Patients with hospital-acquired influenza may present without classical features of an influenza-like illness and this should promote earlier diagnostic testing and isolation to limit spread. Movement of patients after diagnosis is likely to facilitate spread within the hospital.

## Background

Influenza virus seasonally affects approximately 5–10% of the world’s population [1]. The spread of influenza through the hospital setting is thought to be facilitated by healthcare workers, other patients and visitors, resulting in rates of hospital-acquired influenza between 3 and 24% [2–6]. Acquisition of influenza virus through the hospital setting has been associated with increased morbidity, mortality and healthcare costs [5, 7–9]. In 2017, Australia experienced its highest levels of influenza virus activity since the 2009 H1N1 pandemic [10]. The severity of the 2017 influenza season in Australia was associated with a poor overall vaccine efficacy estimated between 33 and 36% and efficacy against the H3N2 strain of 10–25% [11]. Where previous studies of hospital-acquired influenza have examined general trends over multiple seasons [3, 5–7], we aimed to conduct a more focused and detailed single-centre examination of the characteristics, management and outcomes of patients with hospital-acquired influenza. This included factors such as bed location and bed moves which have not been investigated previously.

## Methods

### Design

A surveillance based study was conducted on patients with laboratory-confirmed influenza at the Canberra Hospital from 6th April 2017 to 24th October 2017. Canberra Hospital is a 620 bed tertiary referral hospital and the largest in-patient facility in the Australian Capital Territory (supporting a population of almost 540,000). The hospital caters for all specialties, with majority of the wards in a single, ten-storey building, configured with one, two and four bed rooms. As a participant hospital in the Australian Influenza Complications Alert Network (FluCAN), data is collected via chart review and patient interview during each influenza season [12]. All hospitalised adults (≥18 years) who tested positive for influenza via real time PCR testing from any respiratory sample were included in the study. Baseline demographics and outcomes for hospitalised patients with influenza from the 2017 FluCAN dataset was supplemented with additional data collected retrospectively using hospital information systems. Hospital-acquired influenza was defined as symptom onset ≥48 h (based on the average incubation time of 1.4 days for influenza [13]) after an admission to the study hospital unrelated to respiratory illness. An acute onset of symptoms including fever ≥38 °C in addition to a cough or sore throat was defined as influenza-like-illness (ILI), and non-ILI being a presentation missing one or both of the ILI criteria. Where symptom onset was not known, a laboratory diagnosis of influenza ≥7 days after admission was defined as a hospital-acquired case. Cases with unknown symptom onset and a diagnosis < 7 days after admission required further analysis of patient files. All other influenza cases were defined as community-acquired. Although the definition of hospital-acquired influenza is non-standardised from the perspective of delay between admission and diagnosis [14], we adopted the lower threshold of 48 h which would have the outcome of decreasing effect size and potentially underestimating differences between the groups.

Focused analysis on hospital-acquired patient bed placements, bed moves and other hospital-acquired influenza patient contacts were limited to the period of 4 days prior to symptom development due to the incubation period of influenza ranging 1–4 days [15]. A bed move refers to a patient transfer to another bed within the hospital.

### Statistical analysis

Continuous variables were tested for normality and were analysed with the Mann-Whitney U test as none fulfilled the criteria for normality. Categorical variables were analysed using the *χ*^2^ test or Fisher’s exact test where appropriate. We calculated crude odds ratios for outcomes of patients with community and hospital-acquired influenza, with community-acquired influenza as the reference group. These were reported with 95% confidence intervals and *P-*values ≤0.05 were considered statistically significant. We reviewed age distributions of the two groups and given the similarity between the two we did not proceed to multivariate analysis controlling for age. Statistical analysis was performed using IBM SPSS Statistics 22 software.

Ethics approval for the study was obtained from the ACT Health Human Research Ethics Committee (Protocol number ETHLR1.10.105).

## Results

### Influenza diagnosis

A total of 292 patients with laboratory confirmed influenza were included in the study, 28 (9.6%) of whom were hospital-acquired cases and 264 (90.4%) community-acquired. This proportion of hospital-acquired influenza patients at this institution was considerably greater than all of the 5 years prior, which ranged from 0.9 to 5.8% and employed the same definition of hospital acquisition (FluCAN investigators, unpublished data). The attack rate of hospital-acquired influenza was also greater in 2017 than in the previous year, with 28 cases in 16,112 admissions (0.17%) vs. 11 in 17,733 (0.06%), respectively.

**Fig. 1:**
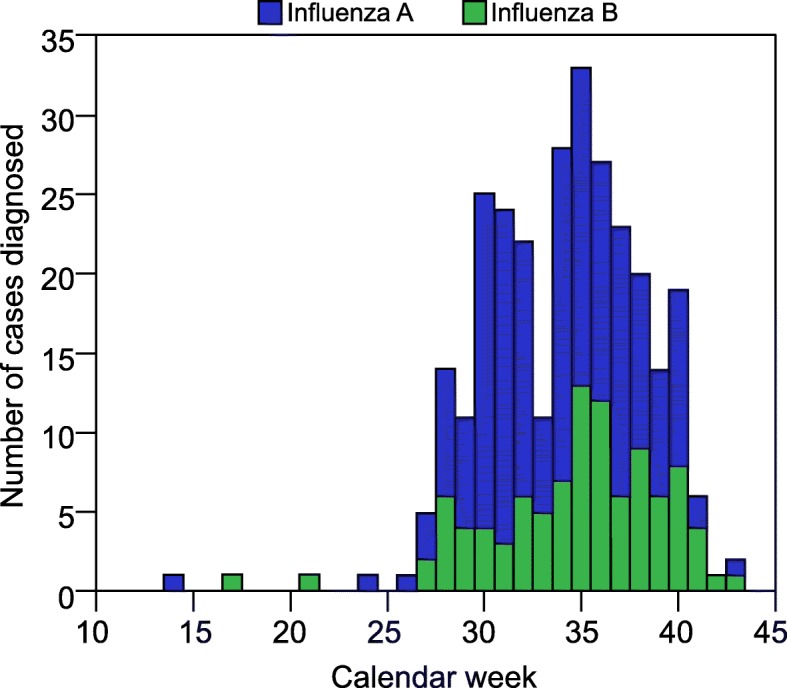
Number of influenza **a** and **b** diagnoses per calendar week in 2017

**Fig. 2:**
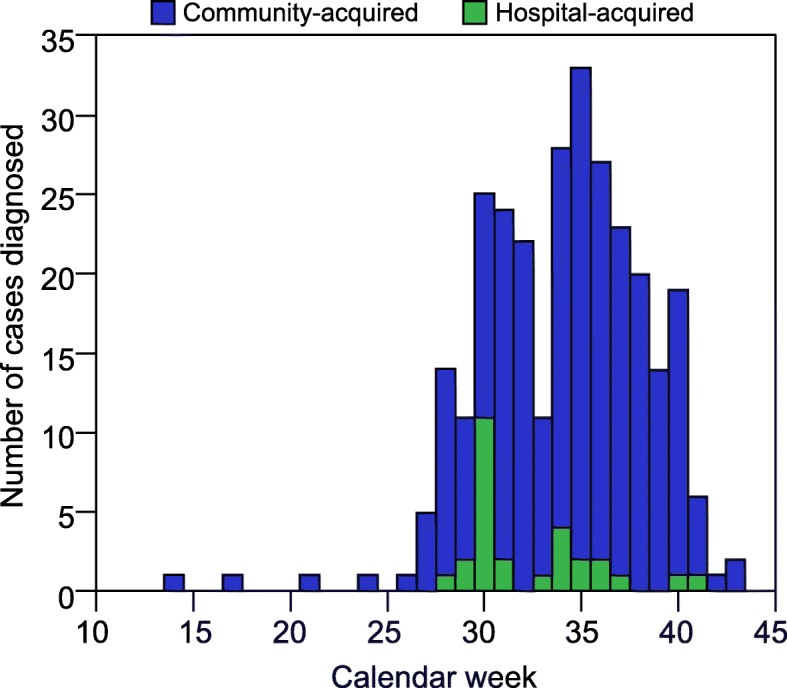
Number of community and hospital-acquired influenza diagnoses per calendar week in 2017

The overall cases occurred in two peaks - centred around calendar weeks 30 and 35, corresponding to two peaks for influenza admissions (Fig. 1). The majority of cases were due to influenza A (193, 66.1%) with over 90% of the 29 patients who were subtyped having H3N2. Compared to the activity of community-acquired influenza which followed a traditional epidemiological curve, there were three bursts of hospital-acquired cases over the season suggesting three sustained episodes of intra-hospital transmission (Fig. 2). Hospital-acquired cases were significantly more likely to have influenza A (*p* = 0.021).

### Patient characteristics

Table 1 compares the characteristics between community and hospital-acquired influenza. Baseline characteristics did not differ significantly between the two groups. There was no significant overall difference between groups in terms of having a comorbidity (92.9% vs. 89.2%) yet hospital-acquired patients were significantly more likely to have diabetes, a malignancy or chronic liver disease (*p* = 0.015, 0.046 and 0.043 respectively). A lower proportion of the hospital-acquired group were vaccinated for influenza in the 2017 season compared with the community-acquired group (41.7% vs. 60.1%). Vaccination rates among healthcare workers at the hospital were reported to be 50%.

**Table 1:** Overview of patient characteristics for community and hospital-acquired influenza

Characteristic	No. (%) patients		
	Community-acquired influenza^‡^	Hospital-acquired influenza^§^	*p* Value
Number of patients	264	28	–
Age, median [IQR]	76 [62–84]	79 [62–90]	0.624
Female sex	142 (53.8)	10 (35.7)	0.069
Indigenous	4 (1.5)	0	1
Chest X-ray taken	236/259 (91.1)	26 (92.9)	1
Consolidation present on chest X-ray	63/236 (26.7)	5/26 (19.2)	0.41
Influenza type			
Influenza A	169 (64.0)	24 (85.7)	0.021
Influenza B	95 (36.0)	4 (14.3)	0.021
Influenza vaccine in 2017	66/97 (60.6)	5/12 (41.7)	0.106
Clinical presentation			
ILI	170 (64.4)	7 (25.0)	< 0.001
Non-ILI	57 (21.6)	21 (75.0)	< 0.001
Pneumonia ± ILI	37 (14.0)	0	0.033
Any comorbidity	214 (89.2)	26 (92.9)	0.191
Chronic respiratory disease	104/254 (40.9)	5/27 (18.5)	0.023
Diabetes	57/257 (22.2)	12 (42.9)	0.015
Malignancy	37/255 (14.5)	8/26 (30.8)	0.046
Chronic liver disease	11/256 (4.3)	4/27 (14.8)	0.043
Immunosuppression	24/257 (9.3)	4/27 (14.8)	0.321
Cardiac disease	94/257 (36.6)	14 (50.0)	0.164
Obesity	31/154 (20.1)	4/22 (18.2)	1
Neurological disease	58/258 (22.5)	10/26 (38.5)	0.069
Chronic renal disease	36/258 (14.0)	4/27 (14.8)	1
Nursing home	41/262 (15.6)	4/27 (14.8)	1
Pregnancy	12/142 (8.5)	1/10 (10.0)	1
Smoking			
Non smoker	60/161 (37.3)	6/16 (37.5)	0.985
Past smoker	78/161 (48.4)	7/16 (43.8)	0.72
Current smoker	23/161 (14.3)	3/16 (18.8)	0.709

NOTE: *ILI* Influenza-like illness, *IQR* Interquartile range. ^‡^ Denominator is 264 unless otherwise specified. ^§^ Denominator is 28 unless otherwise specified

The clinical presentation between the two groups varied considerably. The community-acquired group mostly presented with an ILI or pneumonia with or without ILI (*p* < 0.001, *p* = 0.033 respectively), and the hospital-acquired group presented mostly with a non-ILI (*p* < 0.001). Onset of symptoms for patients with hospital-acquired influenza was a median of 13 days after admission.

### Patient outcomes

Table 2 compares the management and outcomes related to acquisition of influenza. Patients with hospital-acquired influenza were diagnosed sooner after symptom onset compared with community-acquired influenza (*p* < 0.001). When considering time from symptom onset to treatment, 62.5% of hospital-acquired cases were treated within 48 h compared with only 39.8% of community-acquired cases (*p* = 0.033), with a median duration to treatment of 2 days and 3 days respectively (*p* = 0.022). During hospital stay, 10.7 and 14.8% of patients with hospital-acquired and community-acquired influenza, respectively, were admitted to the intensive care unit (ICU). The median length of stay in the ICU did not differ between the two groups.

**Table 2:** Comparison of management and outcomes associated with community and hospital-acquired influenza

Variable	No. (%) patients			
	Community-acquired influenza^‡^	Hospital-acquired influenza^§^	*p* Value	Odds ratio (95% CI)
Days from symptom onset to admission, median [IQR]	3 [2–5]	na	–	–
Days from admission to symptom onset, median [IQR]	na	13 [4–32]	–	–
Days from symptom onset to diagnosis, median [IQR]	3 [2–5]	1 [1–2]	< 0.001	–
Received antivirals (oseltamivir)	233/250 (93.2)	24/27 (88.9)	0.426	0.58 (0.16–2.14)
Days from symptom onset to treatment, median [IQR]	3 [2–5]	2 [1–3]	0.022	–
Received antivirals within 48 h of symptom onset	82/206 (39.8)	15/24 (62.5)	0.033	2.52 (1.05–6.02)
ICU admission	39 (14.8)	3 (10.7)	0.778	0.69 (0.20–2.40)
Length of ICU stay, median days [IQR]	4 [3–6]	4 [4–4]	0.687	–
Length of hospital stay, median days [IQR]	5 [2–9]	38 [18–66]	< 0.001	–
Days from diagnosis to discharge, median [IQR]	5 [3–9]	13 [6–33]	< 0.001	–
30 Day outcome				
Still hospitalised	9/262 (3.4)	8 (28.6)	< 0.001	11.24 (3.91–32.31)
Discharged	237/262 (90.5)	18 (64.3)	0.001	0.19 (0.08–0.46)
Readmitted for same illness	5/262 (1.9)	0	1	–
Death in hospital (all cause)	11/262 (4.2)	2 (7.1)	0.363	1.76 (0.37–8.35)

NOTE: *CI* Confidence interval, *ICU* Intensive care unit, *IQR* Interquartile range, *na* not applicable. ^‡^ Denominator is 264 unless otherwise specified. ^§^ Denominator is 28 unless otherwise specified

Overall, the hospital-acquired group had a significantly longer length of stay in hospital than the community-acquired cases (38 vs. 5 days, *p* < 0.001). They also had a significantly longer length of stay after influenza diagnosis (13 vs. 5 days, *p* < 0.001). Two (7.1%) hospital-acquired influenza patients died within 30 days of diagnosis, which was not significantly different compared to the 11 (4.2%) community-acquired patients.

### Infection control

**Fig. 3:**
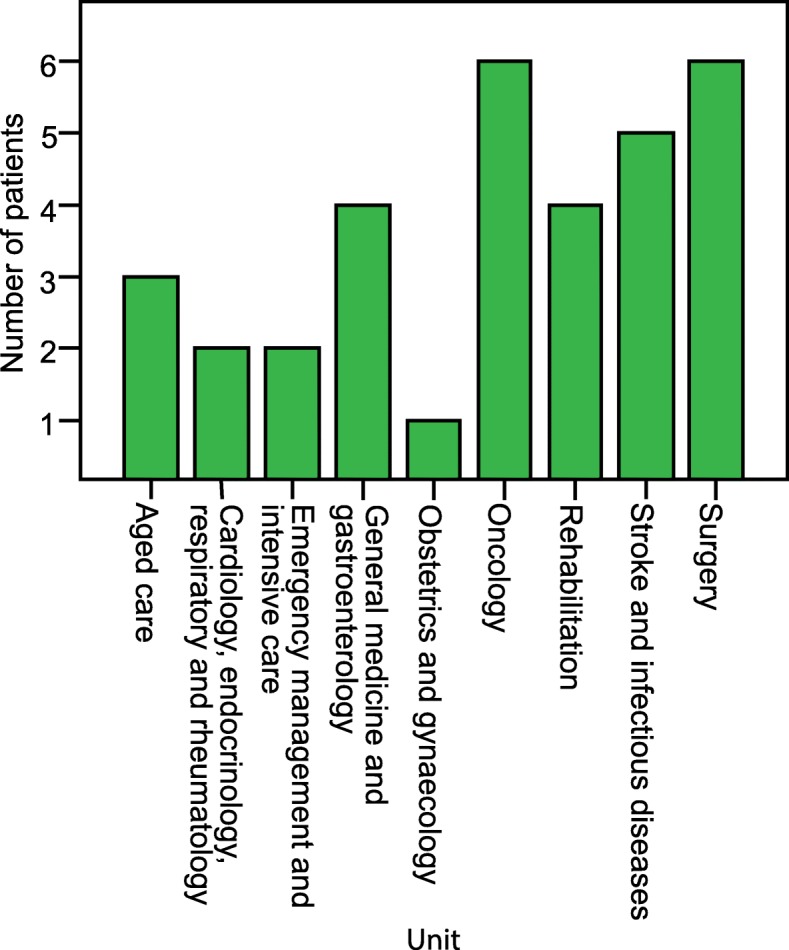
Hospital-acquired influenza cases present in units during patient incubation period

The median number of bed moves during hospitalisation for the hospital-acquired group was 5, however most of these moves took place outside of the incubation period. After diagnosis 8 patients were moved at least once, with one patient moved 6 times. Twenty two hospital-acquired patients were in multiple-occupancy rooms during their incubation period, 9 of whom were in quadruple occupancy rooms. Of the 28 patients who acquired influenza in hospital 17 shared a ward with another patient who was also classified as a hospital-acquired case during this period, suggesting intra-hospital transmission. A single patient was exposed to four other hospital-acquired cases during their incubation period. Figure 3 shows the number of hospital-acquired cases that were present in each unit during the patient’s incubation period. The oncology and surgery units had the highest number of patients with hospital-acquired influenza. After diagnosis 19 (68%) of the hospital-acquired patients were moved into single room isolation however the remaining 9 (32%) patients were moved into double rooms with full occupancy, 7 of which were placed with non-hospital-acquired influenza patients.

## Discussion

This study showed a higher burden of hospital-acquired influenza compared with recent years. The proportion of influenza patients that had hospital acquisition in the 2017 season (9.6%) was considerably greater than all of the 5 years prior in the same hospital. The number of hospital-acquired cases in the single season, 28 (9.6%), exceeded that of a previous Australian multi-centre, multi-season study using FluCAN data, which was 26 (4.3%) cases [5]. This allowed for a more detailed analysis than has previously been conducted of important infection, prevention and control variables including point of acquisition, bed moves, vaccination rates and clinical characteristics of disease.

**Fig. 4:**
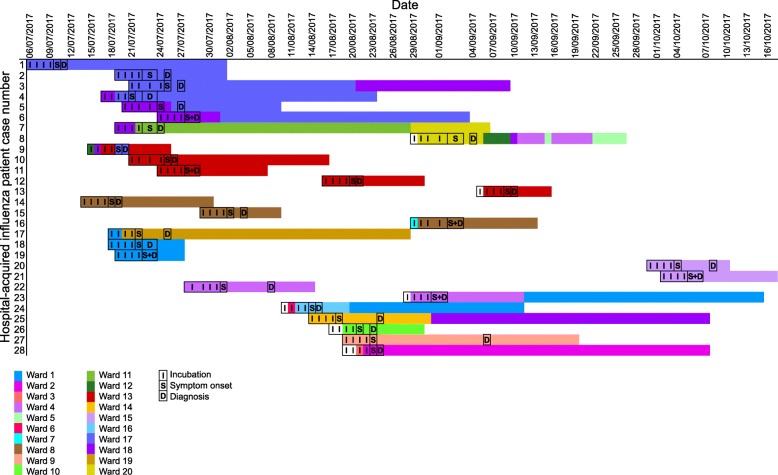
Ward placement of hospital-acquired influenza patients during incubation period, symptom onset, diagnosis and maximum 45 days following diagnosis. Note: an uncoloured ‘**I’** indicates the patient was not in hospital on this day. 45 days following diagnosis was chosen as the limit of the inclusion period due to the maximum documented influenza viral shedding period in immunocompromised patients of 44 days [20]

Hospital-acquired cases mostly occurred in clusters in terms of both date of diagnosis and bed location. This was in contrast with a previous observational study which observed a random scattering of patients across different wards of the hospital, suggesting that hospital-acquired influenza is randomly and irregularly introduced [3]. Influenza spread in hospital may originate from healthcare workers, patients and visitors. Multiple occupancy rooms offer greater opportunities for spread through these contacts, with one study comparing single with double rooms and observing higher hospital-acquired influenza risk in the double rooms [16]. In our study, 22 of the hospital-acquired cases shared a room with at least one other patient during their incubation period. It was also observed that, during the incubation period, 17 of the hospital-acquired cases also shared a ward with at least one other patient who acquired influenza in hospital. While one study identified a single cluster of hospital-acquired cases during two influenza seasons, patient location was only noted at the time of diagnosis [3]. Our study mapped the ward location of the hospital-acquired cases during the incubation period, time of diagnosis and post diagnosis. Figure 4 demonstrates the overlap in location of patients with hospital-acquired influenza during these periods. Although the incubation period can range from 1 to 4 days [15], infectivity in most adults begins approximately 1 day before symptom development to 6–7 days post [17]. Though it is possible that the clusters of cases observed were introduced by an index visitor or staff member, the data is at least suggestive of intra-hospital transmission. Putative transmission exists between patients 2, 3, 4 and 9 on ward 17; between patients 9, 10 and 11 on ward 6; and between patients 18 and 19 on ward 1. The intra-hospital transfer of patient 9 is an example of how a bed move may have facilitated the transmission of influenza in the hospital. Ideally the bed locations of all 292 patients with influenza should be examined to get a true picture of the hospital epidemiology, however this was beyond the scope of this study.

The oncology unit had 6 hospital-acquired cases during their incubation period, 4 of which were immunocompromised. Viral shedding is prolonged in immunocompromised patients [2, 18, 19], with influenza virus documented to be present for up to 44 days in immunocompromised adults [20]. If respiratory precautions and isolation are ceased too early this may result in prolonged spread of virus. Although the majority of cases were moved into single rooms for appropriate isolation, the policy adherence was incomplete, with 9 of the patients (all with at least one significant comorbidity) moved into double occupancy rooms, 2 of which were placed in the same room. This provided further opportunity for intra-hospital spread of influenza. Due to the high demand for single rooms during the study period, itself a reflection on the high rate of admissions for influenza, adhering to isolation policy is occasionally unachievable. Overall, the median number of bed moves for hospital-acquired patients was 5, an issue which has not been investigated in previous observational studies. Patients who have been transferred from another ward in a hospital have been found to have an increased risk of acquiring an infection, with this risk estimated to be 2.5 times higher than a non-transferred patient [21]. Although the bed moves in this study were mostly outside the incubation period, bed moves post influenza acquisition could have facilitated influenza spread to other patients and staff.

Intra-hospital transmission of influenza places a significant yet largely preventable burden on the healthcare system and on individual patients. Patients with hospital-acquired influenza have a significantly longer length of stay compared with those who acquire influenza through the community, which is a major driver of increased costs due to hospital-acquired infections [22]. During this longer length of stay there is a greater window of opportunity for viral transmission to other patients and staff, therefore becoming a source of hospital-acquired influenza themselves. This is particularly relevant to immunocompromised patients who have a prolonged period of viral shedding. Our data indicating clusters of spread suggest that intra-hospital transmission is likely, and hospital-acquired influenza is not simply introduced from members of the community attending the hospital as staff or visitors.

Patients who acquired influenza in hospital mostly presented with the absence of one or both criteria that define ILI. This result likely reflects the high index of suspicion created by a high burden of community-acquired disease. It is possible that during less active seasons atypical presentations remain undiagnosed. Furthermore, as influenza testing is performed at the physician’s discretion after a suspicion of ILI, this atypical presentation may result in an under-diagnosis of hospital-acquired influenza. Both factors suggest that the relative proportion of hospital-acquired cases may be more than this study and others have reported. Both primary care and hospital-based clinicians should be alert to the possibility of a febrile illness without respiratory symptoms being influenza and that atypical presentations, for example gastro-intestinal illness, can occur.

The median period (1 day) between symptom onset and diagnosis of hospital-acquired influenza suggests that when symptoms were identified in-hospital testing was performed rapidly and appropriately. Any acute development of symptoms in admitted high-risk hospital patients during the influenza season, whether it is ILI or non-ILI, should be approached with high suspicion that hospital-acquired influenza could be a potential diagnosis.

The majority of the hospital-acquired and community-acquired groups received antiviral treatment (88.9 and 93.2% respectively) and hospital-acquired influenza was treated sooner than community-acquired influenza. The benefits of antiviral treatment are best achieved when initiating within 48 h after symptom onset [23]. The proportion of patients receiving treatment within this window was low (62.5% of hospital-acquired cases and 39.8% of community-acquired cases) thus potentially impacting outcomes of patients. Although it is difficult to commence treatment early for community-acquired cases due to a delay between symptom onset and hospital admission, no such delay should exist for hospital-acquired cases. Furthermore, early treatment with oseltamivir may decrease the infectivity of the individual [24]. In the hospital setting oseltamivir treatment should be part of a bundle to limit ongoing spread as an adjunctive measure to isolation, hand hygiene and other infection control measures. As our institution has a policy of early treatment with oseltamivir, this study has provoked ongoing audit-based and qualitative research into the barriers for early prescribing of antivirals in hospital-acquired influenza.

In contrast to previous observational studies which found hospital acquisition to be associated with an older population [3, 6, 7], we found no statistical correlation. As aforementioned, the 2017 influenza vaccine efficacy was low, in particular against influenza A(H3N2) [25]. Of the 12 hospital-acquired patients with known vaccination status, only 5 were vaccinated. Although the vaccination status was unknown for many patients, both the community and hospital-acquired groups had high rates of comorbidities (89.2 and 92.9%, respectively), all of which are indications for influenza vaccination [26]. If the influenza strain match had been effective for the 2017 virus, vaccination may have reduced the number of cases.

Hospital-acquired influenza has been associated with increased morbidity and mortality [5, 7, 9], however our results found little difference between the two groups when assessing ICU admission and 30 day all-cause mortality. A marked increase in length of stay was observed in the hospital-acquired group, an effect which persisted even after subtracting the days of admission prior to infection. The magnitude of this finding is likely confounded by the severity of the patients’ initial presenting complaint and the time taken for the appropriate management, though it does represent an increased burden on hospital occupancy during the influenza season.

Limitations of this study should be taken into account when interpreting the results. Focussing the epidemiologic analysis on the cohort of hospital-acquired influenza patients means that true transmission pathways could not be ascertained. Future studies may employ a wider sampling strategy and the use of radio-frequency identification [27] or genomics, for example whole genome sequencing [28], to validate transmission pathways. Our use of a threshold of 48 h to define hospital-acquired influenza may have led to a misclassification of some community-acquired cases as hospital-acquired, potentially leading to differences in some of the variables not reaching statistical significance.

## Conclusion

The magnitude of the influenza season in 2017 resulted in unusually high numbers of hospital-acquired influenza and an opportunity to study this issue in-depth in a single centre. The cluster pattern of intra-hospital transmission suggests the need for increased infection prevention and control, and further research into the role of healthcare workers, patients and visitors in the hospital transmission of influenza.

## Abbreviations

CI: confidence interval
ICU: intensive care unit
ILI: influenza-like illness
IQR: interquartile range
